# Characterization and antifungal activity of a plant peptide expressed in the interaction between *Capsicum annuum* fruits and the anthracnose fungus

**DOI:** 10.1042/BSR20192803

**Published:** 2019-12-20

**Authors:** Álan Chrisleyr Maracahipes, Gabriel Bonan Taveira, Lorran Yves Sousa-Machado, Olga Lima Tavares Machado, Rosana Rodrigues, André Oliveira Carvalho, Valdirene Moreira Gomes

**Affiliations:** 1 Laboratório de Fisiologia e Bioquímica de Microrganismos, Centro de Biociências e Biotecnologia, Universidade Estadual do Norte Fluminense Darcy Ribeiro, Campos dos Goytacazes, RJ, Brazil; 2 Laboratório de Quimica e Função de Proteínas e Peptídeos, Universidade Estadual do Norte Fluminense Darcy Ribeiro, Campos dos Goytacazes, RJ, Brazil; 3 Laboratório de Melhoramento e Genética Vegetal, Centro de Ciências e Tecnologias Agropecuárias, Universidade Estadual do Norte Fluminense Darcy Ribeiro, Campos dos Goytacazes, RJ, Brazil

**Keywords:** Anthracnose, Antifungal, Antimicrobial Peptides, Fungi, Pepper

## Abstract

Plant defensins are low molecular weight basic peptides ranging from 5 to 7 kDa, with capacity of inhibiting various pathogens, including fungi. They are present in different tissues of plants, including floral parts and fruits of *Capsicum* sp. The IIF48 extract, present in immature fruits of *Capsicum annuum* inoculated with *C. gloeosporioides*, was able to inhibit up to 100% growth ‘*in vitro’* of the fungus *Colletotrichum gloeosporioides*. The main objective of this work was the purification and antifungal activity characterization of a defense-related plant defensin-like isolated of the IIF48 immature fruits extract. The IIF48 extract was subjected to HPLC purification and 13 fractions were obtained, followed by a tricine gel electrophoresis to obtain the protein profile. The different fractions were submitted to a growth inhibition assay against *C. gloeosporioides* fungus. Fraction 7 (F7) was the most active causing 73% inhibition. Because of the higher F7 activity and the presence of only a peptide of approximately 5 kDa this fraction was subjected to N-terminal sequencing. F7 fraction was carried out plasma membrane permeabilization assays, induction of intracellular ROS production analysis and investigated mitochondrial membrane potential. The F7 fraction showed significant inhibitory activity on the tested fungus, besides promoting membrane permeabilization, induction of endogenous ROS production in *Colletotrichum* cells and impairing mitochondrial functionality. The first 18 amino acid sequence of the F7 fraction peptide suggests homology to plant-like defensin and was named IIFF7Ca. We also concluded that IIFF7Ca peptide has an effective antimicrobial action against the fungus *C. gloeosporioides.*

## Introduction

Plant defensins are basic low-molecular-weight peptides ranging from 5 to 6 kDa, have 45 to 55 amino acid residues, with eight conserved cysteine residues linked by four disulfide bonds that ensure increased strength and structural stability. Studies have shown that plant defensins have inhibitory activity against fungal species of the genera *Colletotrichum, Fusarium, Aspergillus, Alternaria, Nectria* and *Candida*, among others. The inhibitory activity and the concentration required for inhibition were dependent upon the specific fungus and also on the defensin [1]. Several of the plant defensins showed a constitutive expression pattern with up-regulation following pathogens attacks, and different biotic or abiotic stresses. Defensins are found and identified in different organ plants, like seeds, leaves, tubers, flowers, pods and fruits [1,2].The first experiments done to try to unravel the mechanism of action of the plant defensins were conducted by Thevissen et al. [3]. The present study verified that the interaction of the defensins with the fungus *Neurospora crassa* resulted in membrane potential changes, and the concomitant alkalinization of the incubation medium [3]. Defensins may also interact with some components of the plasma membrane and have the ability to internalize in the cell through receptors. Consequently, the production of reactive oxygen species (ROS) caused by defensin can lead to pathogen cell death. The defensins present toxicity to some microbial cells and are therefore of interest in the production of new antimicrobial agents for both medicinal and biotechnological purposes [4].

Seo et al. [5] and Meyer et al. [6] identified defensins in various *Capsicum* tissues, including the floral parts and fruits at different stages of maturation. The appearance of defensin in pepper fruits was related to fruit ripening or induced by wounds and/or pathogen attacks. The promoters of genes encoding defensins had in their sequence transcription factors binding sites related to the signaling of jasmonic acid and ethylene, and overexpression of the gene occurred mainly in immature fruits of *Capsicum* through the exogenous application of methyl jasmonate. A defensin, designated as J1-1, has been described in the fruit of bell pepper and their functional characterization was also conducted to evaluate its biotechnological potentiality in transgenic pepper plants against the causal agent of anthracnose disease, *Colletotrichum gloeosporioides* [5].

In a previous work, we observed differences in the protein profile of the crude extract of immature fruits of the *C. annuum*, accession UENF1381, 48 h after inoculation with the fungus *C. gloeosporioides* (named IIF48). IIF48 was able to inhibit 100% of the growth of the fungus *C. gloeosporioides* at 200 μg ml^−1^, and by subjecting a 5 kDa band present in this sample, to sequencing by mass spectrometry, the sequence obtained showed similarity to defensins [7]. Based on this information, the intention of this work was to purify the previously identified defensing. However, the results presented in this work suggested we had purified another new peptide in the IIF48 that also belongs to the plant defensin family. Additionally, we had analyzed the mechanism of action of this new defensin on the fungus *Colletotrichum gloeosporioides.*

## Materials and methods

### Obtaining and infection of fruits

The study was conducted at Universidade Estadual Norte Fluminense Darcy Ribeiro, Campos dos Goytacazes, Rio de Janeiro, Brazil. Immature fruits of *C. annuum* of the access UENF1381, resistant to fungus *C. gloeosporioides*, were harvested 30 days after the anthesis, and taken to Laboratório de Fisiologia e Bioquímica de Microrganismos (LFBM) where they were disinfestated by sequentially immersion in alcohol 70%, sodium hypochlorite 0.5% and ultrapure water. After disinfestation, an injury was made in the middle region of each fruit using a sterilized needle, where a drop of 20 μl of *C. gloeosporioides* spore solution (10^6^ conidia/ml) was inoculated. The fruits were packed in a humid chamber and after 48 h, the peduncle and the seeds were removed, and the fruits were submitted to protein extraction [7].

### Obtaining the IIF48 extract

Protein extraction was performed according to Taveira et al. [8]. Briefly, 40 g of fruit pulp (without seeds and stem) was processed into 200 ml of phosphate buffer for 15 min with a multiprocessor and the extraction was placed at 4°C for 2 h with shaking. The extraction was centrifuged for 45 min at a temperature of 4°C and a rotation of 15,400 × *g*, and then it was filtered through a paper filter and the insoluble sediment was discarded. The ammonium sulfate at 70% saturation was added to the crude extract and stirred for 40 min at 4°C overnight. Next day, the solution was centrifuged for 45 min at 4°C and 15,400 × *g*, and the supernatant was discarded. The precipitate was resuspended and placed in a water bath for 15 min at 80°C and then centrifuged for 30 min at 15,400 × *g*. The precipitate was discarded and the supernatant stored [7].

The samples were dialyzed with semipermeable membranes (exclusion cutoff 1000 Da) at 4°C for a period of 3 days with distilled water, with three daily water exchanges. After this period, the samples were concentrated by lyophilization, resuspended in water, conditioned in microcentrifuge tubes and stored at −18°C. Quantitative determinations of protein were done by the Bradford method [9] with bovine serum albumin as standard.

### Peptides purification in HPLC chromatography

The IIF48 sample was subjected to reverse phase chromatography using the C18 column (Pre-Packed Column RT 250-4) (MERK) for crude extract. The run had duration of 100 min, with flow of 0.4 ml/min, being injected 0.5 ml of filtered sample at the beginning of the run. The column was initially equilibrated only with solvent A (H_2_O with 0.1% TFA) (Sigma-Aldrich) for the first two min. From 2 to 90 min there was the addition of a gradient of solvent B (100% propanol (Sigma-Aldrich) in 0.1% TFA) at a concentration of 1–40%. Between 90 and 94 min, the concentration of solvent B increased to 60%. From 95 min onwards the concentration of solvent B was reduced to 0% [8]. After the chromatography, the fractions were diluted in water, lyophilized and had their protein content quantified.

### Electrophoresis in tricine gel

The one-dimensional electrophoresis was performed according to Schagger and Von Jagow [10], where 20 μg of sample was prepared with 5% sample buffer and β-mercaptoethanol (1%), totaling 20 μl. The samples were placed in a water bath at 80°C for 5 min, centrifuged at 15,000 × *g* for 3 min and then applied to each well of the one-dimensional gel. The run was performed with amperage of 400 A, and tension between 22 and 26 V overnight. At the end of the run, the gel was removed from the glass plates and immersed in the Coomassie R dye solution, then in a distain solution; the gel image was recorded with the aid of a gel documentation system.

### Analysis of inhibition of fungal spores growth

The fungus *C. gloeosporioides* was transferred from the stock and placed to grow on Petri dishes containing Sabouraud agar for approximately 15 days at 30°C. After growth, 10 ml of Sabouraud broth was poured onto the plate containing the fungi and the spores were liberated with the aid of a Drigalski loop. This suspension was filtered in gauze to prevent the passage of mycelial debris that could be in solution together with the spores. These spores were then quantified in the Neubauer chamber in the presence of an optical microscope (Axiovison A2, Zeiss). Initially, fungal spores (1 × 10^4^ cells/ml) were incubated in Sabouraud broth containing different concentrations of the fractions obtained from *C. annuum* fruits, and the assay final volume adjusted to 200 µl. The assay was performed on cell culture microplates (96 wells) at 30°C for a period of 24 h. The cell growth was determined by optical density, monitored every 6 h, in a microplate reader at a wavelength of 620 nm. Every test was done in triplicate. The entire procedure was performed under aseptic conditions, in a laminar flow hood, according to a methodology adapted from Broekaert et al. [11].

The statistical analyses were performed using the Graph Pad Prism software (version 5.0 for Windows). The IC50 was calculated based on a linear regression curve, and it was defined as the protein extracts concentration required to inhibit 50% of microorganism growth in the conditions tested.

### N-terminal sequencing by Edman degradation

To obtain the primary structure of peptide of the F7 fraction, 50 μg of protein were lyophilized in a microfuge tube and subjected to Edman degradation sequencing in a Shimadzu PPSQ-23A automatic protein sequencer. The F7 fraction (50 μg) was loaded onto a trifluoroacetic acid (TFA), treated glass fiber membrane and its N-terminal end was sequenced by coupling the peptide with the phenylisothiocyanate (PITC) reagent that reacted with the N-amino terminal residue to form N-phenylthiocarbamoyl (PTC). Then a molecular rearrangement occurs with the cleavage of the first peptide bond, forming the anilinotiazolinone (ATZ) and the remainder of the peptide without the first amino acid. Still in diluted acid medium, there is the conversion of ATZ, to phenylthiohydantoin (PTH) which submited to high pressure reverse phase liquid chromatography. Quantification and identification of amino acids are performed by comparison with a PTH amino acids standard. Sequence alignment was performed in the Blast program, the percentage of sequence identity obtained with the aligned sequences was calculated on the Needleman-Wunsch Global Align Protein Sequences, and the signal peptide of the aligned sequences was determined by the SignalP 4.1 tool. server.

### Effect of fractions on membrane permeabilization

The membrane permeabilization of fungal cells treated with the different peptide fractions present in *C. annuum* fruits was evaluated using the SYTOX Green fluorescent dye, according to the methodology described by Thevissen et al. [3], with some modifications. SYTOX Green is a dye that has high affinity for nucleic acids and penetrates cells only when their membrane is compromised. Immediately after the fungal growth inhibition assay, aliquots of fungal growth were incubated under constant shaking for 15 min at 30 °C with the SYTOX Green fluorescent dye at a final concentration of 0.2 μM according to instructions provided by the manufacturer. The cells were analyzed under an optical microscope (Axiovison A2, Zeiss) equipped with a set of fluorescent filters (excitation wavelength, 450–490 nm; emission wavelength, 500 nm).

### Intracellular ROS induction assay

To evaluate if the mechanism of action of fractions involves the induction of oxidative stress, the fluorescent probe 2′, 7′-dichlorofluorescein diacetate (H_2_DCFDA) was used to measure intracellular ROS, according to methodology described by Mello et al. [12]. After fungal growth inhibition assays, 50 µl of these cells grown in the absence and presence of 100 µg ml^−1^ of the peptide fractions, were incubated with H_2_DCFDA, which is capable of diffusing through plasma membrane of the cells and be hydrolyzed by intracellular esterases to form a non-fluorescent molecule. With increased ROS production, this molecule will react with intracellular ROS forming a fluorescent molecule. After 30-min incubation at room temperature with constant shaking the cells were analyzed under an optical microscope (Axiovison 4, Zeiss) equipped with a set of fluorescent filters (excitation wavelength between 450–490 nm and 500 nm emission). The results represent triplicate experiments.

### Mitochondrial functionality determination assay

Mitochondrial functionality was assessed by fluorescent dye Rhodamine 123 (Sigma-Aldrich). Rhodamine 123 is a cationic fluorescent dye that has high affinity to the membrane electrical potential, thus, it marks active mitochondria in living cells. After the growth inhibition assay, the fungal cells were resuspended and incubated with 10 μg ml^−1^ of Rhodamine 123 under constant shaking at 500 rpm and protected from light for 2 h, and then analyzed by DIC on the optical microscope equipped with a fluorescence filter (excitation wavelength of 506 nm, emission wavelength of 530 nm). The control cells had the same treatment of treated cells with the exclusion of the peptide fractions [13].

## Results

### Purification of a peptide from IIF48 extract

**Figure 1:**
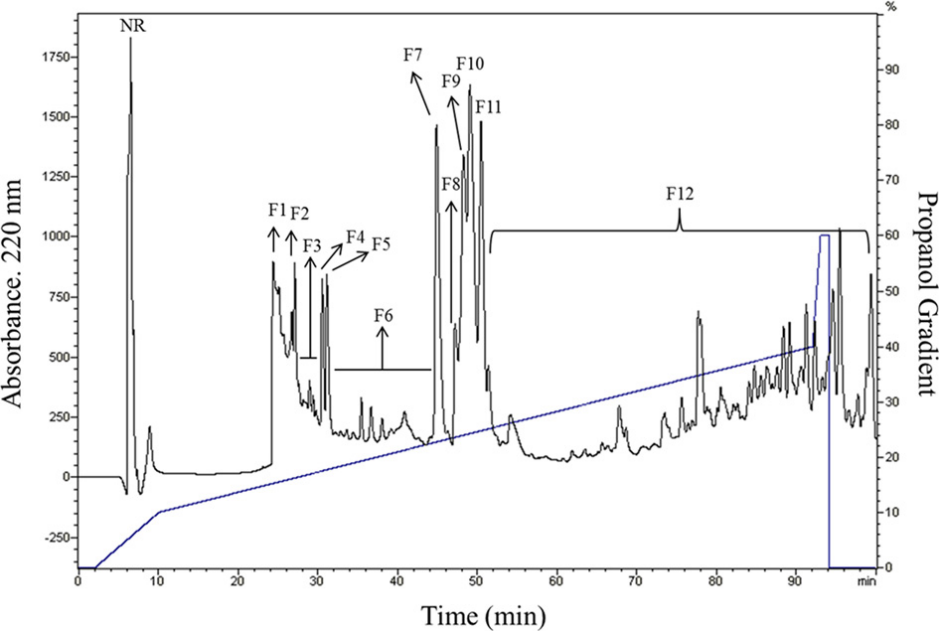
Chromatogram of the IIF48 fraction from fruits of *C. annuum* in reversed-phase The sample IIF48 was separated into 12 fractions, named F1 to F12, and one non-retained fraction (NR). The column was equilibrated and run with 0.1% TFA (Solvent A) and eluted using a gradient (oblique line) of 100% propanol in 0.1% TFA (Solvent B). The flow used was 0.5 mL min^−1^.

**Figure 2:**
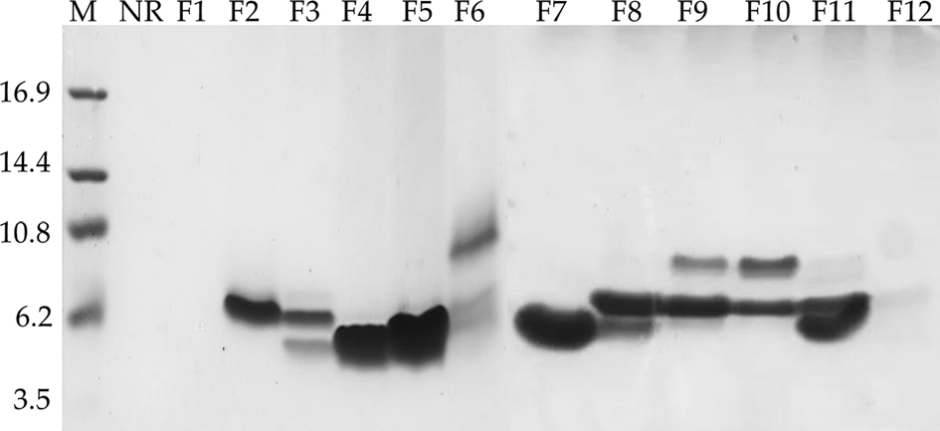
Electrophoretic visualization in Tricine-SDS-PAGE Tricine-SDS-PAGE of the 13 fractions obtained by the fractionation of the IIF48 fraction of *C. annuum* fruit (NR and F1 to F12) by reversed-phase C18 column in HPLC. (NR) non-retained fraction. (M) refers to molecular mass markers in kiloDaltons.

The protein extract IIF48 that was previously described by our research group, presented low-molecular-weight bands ranging from 5 to 11 kDa and presented a band of 5 kDa that did not appear in the control treatment (CIF48 without inoculation) [7]. The IIF48 sample was subjected to reversed-phase chromatography, and after fractionation using a C18 column, 13 fractions were obtained (F1 to F12 fractions and the unretained fraction (NR)) (Figure 1). These fractions were submitted to tricine gel electrophoresis (Figure 2), where several bands with molecular weights between 4 and 10 kDa were visible in almost all fractions except NR and F1. There was a single band marking for fractions F2, F4, F5 and F12 and a major band for the F7 fraction. From the 52 min to the 100 min run, all material was collected together as fraction 12 (F12).

### Growth inhibition assay of the fungus *C. gloeosporioides in vitro*

**Figure 3:**
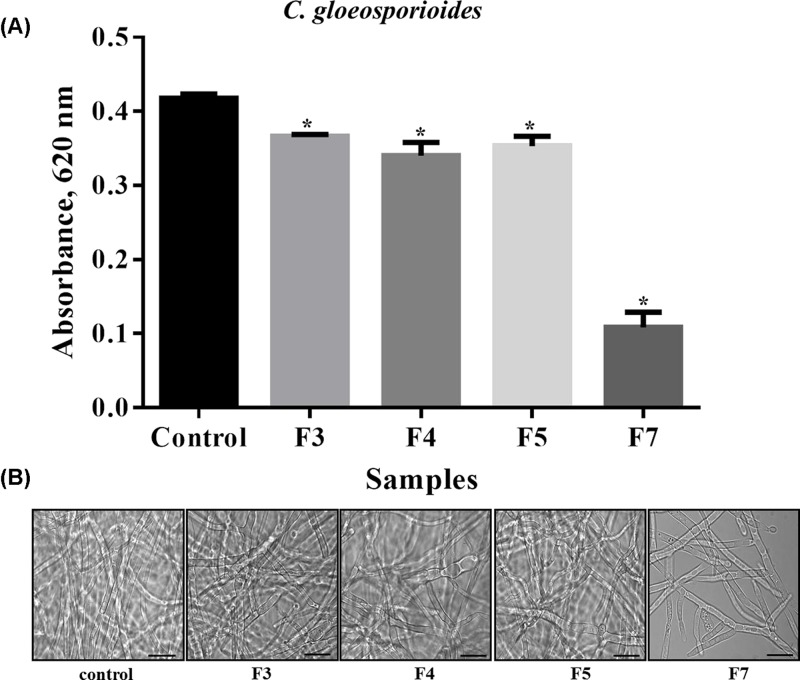
The effect of fractions on the growth of the *Colletotrichum gloeosporioides* (**A**) The effect of F3, F4, F5 and F7 fractions on the growth of the phytopathogen *C. gloeosporioides*. Control (absence of fractions) and 200 µg ml^−1^ of each fraction. (*) indicates significance by the one-way ANOVA test (*P* < 0.05). (**B**) Images of *C. gloeosporioides* cells by light microscopy after 24 h of incubation. Control cells without fractions; bars = 20 µm. Experiments were performed in triplicate.

**Figure 4:**
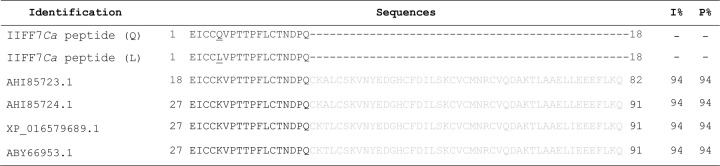
Alignment of amino acid residues from F7 fraction Alignment of the 18 amino acid residues of F7 fraction of 5 kDa obtained by Edman degradation sequencing. Alignment was performed by Clustal Omega, and the sequence obtained was designated IIFF7*Ca* peptide. The sequence obtained showed 94% similarity to the sequences of *Capsicum annuum*: Sequence ID: AHI85723.1 Stress-induced protein 18; Sequence ID: AHI85724.1 Stress-induced protein 19; Sequence ID: XP_016579689.1 flower defensin-like from *C. annuum*; and Sequence ID: ABY66953.1 Thionin-Like. The percentage of identity between the sequences was determined with Needleman-Wunsch Global Align Protein Sequences methods, and the signal peptides were omitted. Q and L forms of peptide correspond to the two different amino acids identified in the same locus, glutamine (Q) and leucine (L), respectively. These amino acid residues were underlined in the sequence. I% – percentage of identity. P% – percentage of positive residues.

A growth inhibition assay against *C. gloeosporioides* with the 13 fractions obtained by HPLC was performed (data not shown), but only four fractions (F3, F4, F5 and F7) showed significant differences in relation to the control (in the absence of peptide fractions) (Figure 3). The F3 fraction showed 12% growth inhibition against *C. gloeosporioides*, the F4 fraction 18.38%, F5 displayed 15.30% growth inhibition, and the most active fraction, F7, inhibited 73.94% of the *C. gloeosporioides* growth at a concentration of 200 μg ml^−1^. Optical microscopy results (Figure 4B) corroborated the results of the inhibition assay, where we clearly observed the greatest reduction in *C. gloeosporioides* hyphae in the presence of the F7 fraction.

### Protein sequencing by Edman degradation

Because of its strongest inhibitory activity, the protein band of the F7 fraction (5 kDa band) was sequenced via Edman degradation. From this analysis a fragment of 18 amino acid residues was obtained (Figure 4). Among the 18 amino acid residues there are three cysteine residues, conserved in position with plant defensins (Figure 4). In position 5 the amino acid gave an ambiguous signal in sequencing – our analysis indicated the possibility of a glutamine (Q) or a leucine (L). The sequence obtained was called IIFF7*Ca* peptide (immature fruit *Capsicum annuum* peptide). In the alignment, the sequence obtained showed a similarity of 94% with sequences already deposited in the NCBI for two proteins induced by stress in *C. annuum*: Stress-induced protein 18 (Sequence ID: AHI85723.1); Stress-induced protein 19 (Sequence ID: AHI85724.1): a protein described as flower specific defensin-like from *C. annuum* (Sequence ID: XP_016579689.1); and a protein described as thionin-like of *C. annuum* with Sequence ID: ABY66953.1. For the alignment, only the mature protein was used, and the percentage of positive amino acids found in the sequences was 94%. Cysteines are conserved in all sequences.

### Mechanisms of action of antimicrobial peptide

**Figure 5:**
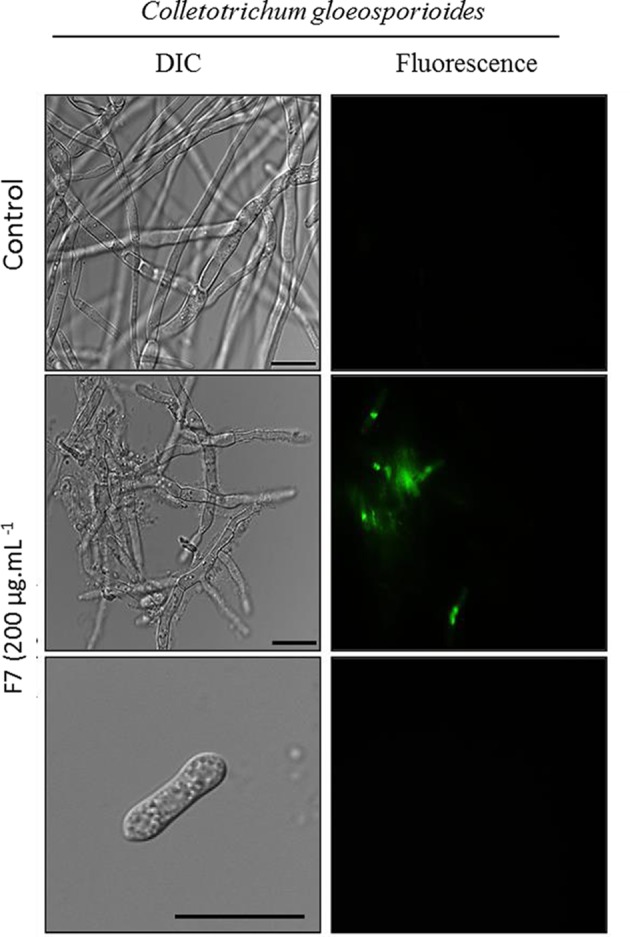
Membrane permeabilization assay Cells and spore of *C. gloeosporioides* by fluorescence microscopy using the fluorescent probe Sytox Green. Cells were treated with 200 µg ml^−1^ of IIFF7*Ca* peptide and then assayed for membrane permeabilization. Control cells were treated only with the Sytox Green probe; bars = 20 µm.

Due to the strong inhibitory activity of the F7 fraction, characterized and named as IIFF7*Ca* peptide, tests for membrane permeabilization was performed for this fraction only. The membrane permeabilization assay (Figure 5) showed that the IIFF7*Ca* peptide was able to cause structural alterations in the plasma membrane of *C. gloeosporioides*, resulting in the permeabilization of fungal cells as indicated by the green fluorescence. It was possible to visualize a few hyphae developing in the presence of the IIFF7*Ca* peptide but deformations were present, such as swollen hyphae and disordered growth. The absence of spores in the controls was also observed, indicating that the spores developed into hyphae; however, it was possible to observe spores in the tests with the IIFF7*Ca* peptide, indicating that the fraction is able to delay the germination process. However, none of the spores observed in the tests showed fluorescence.

**Figure 6:**
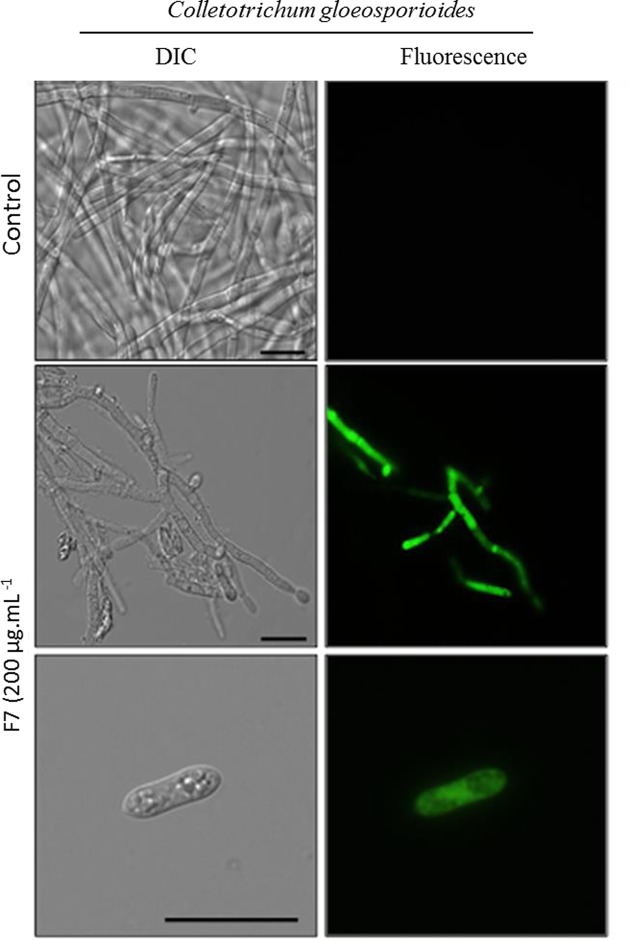
ROS induction assay Cells and spore of *C. gloeosporioides* after ROS induction assay by light microscopy using the fluorescent probe 2,7-dichlorofluorescein diacetate. Cells were treated with 200 µg ml^−1^ of IIFF7*Ca* peptide for 24 h and then assayed for oxidative stress. Control cells were treated only with 2,7-dichlorofluorescein diacetate probe; bars = 20 µm.

**Figure 7:**
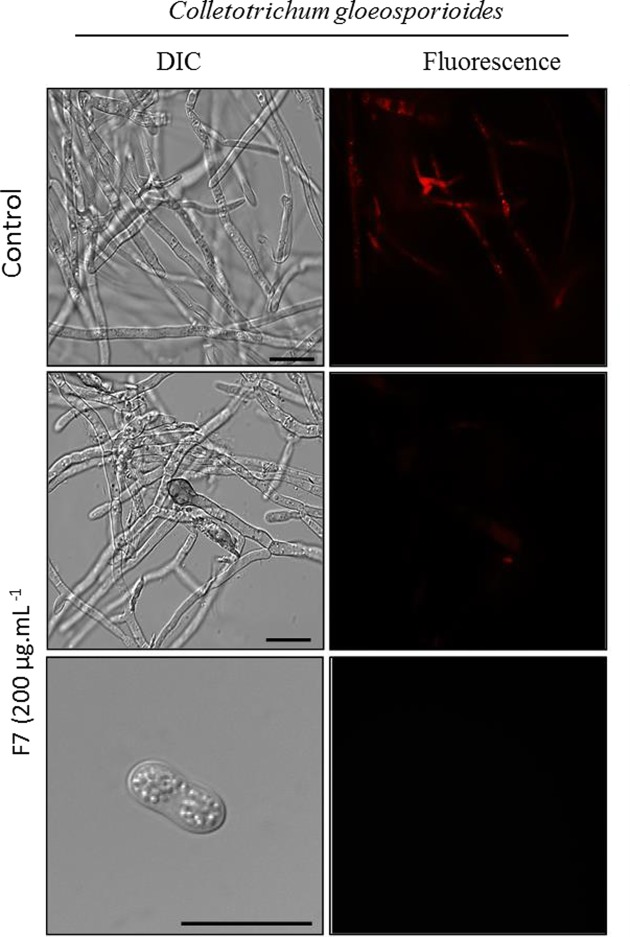
Mitochondrial functionality assay Cells and spore of *C. gloeosporioides* after mitochondrial functionality assay, visualized by fluorescence microscopy using Rhodamine 123 fluorescent probe. Cells were treated with 200 μg ml^−1^ of IIFF7*Ca* peptide for 24 h and then analyzed for mitochondrial functionality. Control cells were treated only with the Rhodamine 123 probe; bars = 20 μm.

An assay was performed to verify whether the IIFF7*Ca* peptide caused the increase in the endogenous production of ROS, where the fungus was incubated along with the fraction for a period of 24 h. In Figure 6, it is possible to verify the fluorescent labeling in both the hyphae and the spores of *C. gloeosporioides*, indicating the increase in the endogenous production of ROS in these structures in the presence of the IIFF7*Ca* peptide, which does not happen in the control (without peptide) (Figure 6). It is suggested that the increase in oxidative stress induced by the IIFF7*Ca* peptide may be the basis of the inhibitory effect against this fungus. Figure 7 shows the assay to verify the mitochondrial functionality of fungal cells. After 24 h of incubation of *C. gloeosporioides* cells with 200 μg ml^−1^ of IIFF7*Ca* peptide, the cells had decreased mitochondrial activity compared to control cells (without peptide), which can be observed by the low fluorescence signal of Rhodamine 123 dye in both treated hyphae and spores. For control cells that have functional mitochondria, a strong fluorescence signal of Rhodamine 123 is observed.

These data show that the IIFF7*Ca* peptide is toxic to *C. gloeosporioides*, causing strong inhibition of cell growth, in addition to causing damage to the few hyphae that have developed and to the spores. It is suggested that this identified fraction and the associated protein may contribute to anthracnose resistance in the immature stage of the fruits of *C. annuum* and are thus important tools to be examined in the improvement of *Capsicum* breeding for resistance to pests and diseases.

## D**iscussion**

The diversity of AMPs in plants is large and so far hundreds of different peptides have been characterized. In our previous work, our group identified a 5 kDa peptide similar to plant defensins in the protein extract, called IIF48, this peptide was named *Ca*Def_1_. Based on these results, the main objective of this work was to isolate and characterize the *Ca*Def_1_; however, our results suggest that another new defensin present in this same protein extract (IIF48) was purified (Supplementary Figure). The presence of these different defensins can be explained by the fact that these peptides are expressed from multigenic family, e.g*.* it was originally demonstrated the description of two genes in *Capsicum annuum* coding for plant defensins and more recently, other studies have revealed that this family is much larger and may comprise up to 300 genes [14–17].

Some studies have shown the isolation of antimicrobial peptides from pepper plants by HPLC chromatography techniques. Santos et al. [18] purified and characterized peptides extracted from seeds of *Capsicum annuum, C. baccatum* and *C. chinense* and obtained a chromatogram with 8 fractions containing peptides with molecular weights between 5 and 10 kDa, for the three species studied. Bard et al. [19] after HPLC chromatography, isolated antimicrobial peptides from *C. baccatum* seeds from seven different fractions that were obtained. One of these fraction called H4 showed similarity with vicilins. Dias et al. [20] performed reversed-phase chromatography of *C. chinense* seeds and obtained three fractions showing peptides with molecular weights between 5 and 8.5 kDa with high inhibitory activity against different yeast growth. Taveira et al. [13], tested the ability of *CaThi*, a thionin-like peptide purified from mature fruits of *C. annuum*, to inhibit the growth of the filamentous fungus *Fusarium solani* and found that the protein inhibited 21, 50 and 83% of fungal growth at concentrations of 12.5, 25 and 50 μg ml^−1^, respectively.

Santos et al. [18] tested the fractions obtained from seeds of *Capsicum* spp. against various filamentous fungi and found that all eight fractions were able to inhibit the growth of *Fusarium lateritium, Fusarium solani* and *Fusarium oxysporum* but were not able to inhibit the growth of *C. gloeosporioides*. Silva et al. [21] tested *CaTI*, a protease inhibitor obtained from *C. annuum* seeds, on the growth of fungi *Colletotrichum lindemuthianum, C. gloeosporioides, F. solani* and *F. oxysporum* and found inhibition against fungi of the genus *Colletotrichum*, but not for those of the genus *Fusarium*. In microscopic analysis, it was concluded that *CaTI* reduced the growth of the mycelium of *C. gloeosporioides*. For *C. lindemuthianum* and *F. oxysporum*, abnormal growth of the hyphae was observed, and for *F. solani*, a cluster of cells was observed in the control treatment. The J1-1/GST defensin of *Capsicum* showed inhibitory activity toward the growth and development of anthracnose fungi, maintaining antifungal activity and interrupting the process of fungal infection of plant tissues [5]. These results confirm the presence of several antimicrobial peptides in *Capsicum*, capable of inhibiting several phytopathogenic fungi of agronomic interest *in vitro*, and the need for further study of these molecules for use in plant breeding for disease resistance. The results obtained in the present work show a fraction with the ability to inhibit the growth of a fungal species that causes anthracnose, one of the most destructive diseases of peppers.

Plant defensins are present throughout the plant kingdom and can trigger several defense-related functions, such as inhibitors of α-amylase enzyme, present mainly in the insect chewing process, and antimicrobial activity against various pathogens [22]. Carvalho and Gomes [1] presented some new characteristics that transgenic plants possess after a superexpression of defensins. Among these characteristics are resistance to heavy metals in *Arabidopsis thaliana*, resistance to fruit and root rot of *Carica papaya*, resistance to *Botrytis cinerea* in *Solanum lycopersicon*, and resistance to early dying disease in *Solanum tuberosum* caused by *Verticillium dahlia*, among other characteristics in different cultures such as sweet taste in *Zea mays*. Van Weerden and Anderson [23] compared the sequences of more than 139 plant defensins. They concluded that although they exhibit a common fold with a β-sheet connected to an α-helix with three sulfide bridges, the overall level of identity between the plant defensins was less than 35% and defensins differed in the activity performed, concluding that their sequences are divergent, which helps to diversify the activities performed by them as antifungal and antibacterial activities, enzymatic inhibitory activities, and important roles in heavy metal tolerance.

About action mechanism involving antimicrobial peptides, Taveira et al. [24], observed plasma membrane permeabilization of *F. solani* cells after 12 h of incubation with 50 μg ml^−1^ of *CaThi*. The presence of propidium iodide after 24 h of incubation was observed, indicating necrotic cell death of some cells. The appearance of fluorescent spots was also observed in the cell nuclei after 48 h of incubation with *CaThi*. That work also showed that after 60 h of incubation, the *CaThi-FITC* peptide was inside the fungal cells. Taveira et al. [13] tested *CaThi* on *Saccharomyces cerevisiae* and *Candida tropicalis* cells and concluded that the protein can induce apoptosis in *S. cerevisiae* cells. Santos et al. [18] showed that the Fa5 fraction of *Capsicum annuum* seeds was able to permeabilize the membrane of the fungi *Fusarium lateritium, F. solani* and *F. oxysporum* after 24 h of incubation with 200 μg ml^−1^ of the fraction. In a study conducted by Seo et al. [5], a large amount of the J1-1 peptide (defensin) was located in the cells invaded by the pathogen and excreted onto the surface of the cell and around the fungus, which shows a broad mechanism of defensin in its utilization by the plant in defense against pathogens. Santos et al. [18] showed that 200 μg ml^−1^ of Fa5 was able to induce ROS in several *Fusarium* species but was not able to induce ROS in *C. gloeosporioides*. Silva et al. [21] found that *CaTI* was able to induce endogenous ROS in all fungi tested at a concentration of 64 μg ml^−1^. According to Camejo et al. [25], ROS act as antimicrobial molecules present in the cell wall that serve to prevent the entry of pathogens. Taveira et al. [13] presented the results of the action of *CaThi* in cells of *C. tropicalis*, where after 24 h of treatment with 10 μg ml^−1^ of the protein, there was a decrease in mitochondrial signals. Aerts et al. [26] evaluated the effects of HsAFP1 antifungal defensin on mitochondrial functionality and ROS production because mitochondria are the main source of endogenous ROS in *Candida albicans* cells. Vieira et al. [27] evaluated the mitochondrial functionality of *C. albicans* in the presence of the *Lp-Def 1* defensin of *Lecythis pisonis* and concluded that there is loss of mitochondrial functionality after 36 h of incubation.

However, as described by Prisi et al. [28] plant defensin mechanisms are more complex than simple membrane permeabilization. Then, the characterization of different mechanisms as interactions with specific lipids, production of reactive oxygen species and effect on mitochondrial activity are very important for find answers about plant defensin action.

## Conclusion

We isolated and characterized a new peptide from *C. annumm* fruits that can be related to plant defense, named IIFF7*Ca* peptide and suggested by Edman degradation sequencing analysis, which belong to plant defensin family. IIFF7*Ca* peptide had antifungal activity against *C. gloesporioides* at low concentrations and toxicity effects causing inhibition of growth, membrane permeabilization, induction of endogenous ROS and decreased mitochondrial functionality of the pathogen. These results indicate that IIFF7*Ca* peptide can be a promising candidate as a potential antifungal molecule to combat pathogens responsible for plant disease and as effective tools in the control of anthracnose.

## Supplementary Material

Supplementary Figure

## Abbreviations

ATZ: anilinotiazolinone
F7: fraction 7
PITC: phenylisothiocyanate
PTC: phenylthiocarbamoyl
PTH: phenylthiohydantoin
TFA: trifluoroacetic acid
