# Supplementary material for: Characterization and antifungal activity of a plant peptide expressed in the interaction between Capsicum annuum fruits and the anthracnose fungus
Source: Biosci Rep. 2019 Dec 20;39(12):BSR20192803. doi: 10.1042/BSR20192803 (PMC6923331; doi:10.1042/BSR20192803)
Supplement: Supplementary Figure [file BSR-2019-2803_supp.pdf]

Characterization and antifungal activity of a plant peptide expressed in the interaction between *Capsicum annuum* fruits and the anthracnose fungus

Álan Chrisleyr Maracahipes[1], Gabriel Bonan Taveira[1], Lorran Yves Sousa-Machado[1], Olga Lima Tavares Machado[2], Rosana Rodrigues[3], André Oliveira Carvalho[1], Valdirene Moreira Gomes[1*]

[1] Laboratório de Fisiologia e Bioquímica de Microrganismos, Centro de Biociências e Biotecnologia, Universidade Estadual do Norte Fluminense Darcy Ribeiro, Campos dos Goytacazes, RJ, Brazil.
[2] Laboratório de Quimica e Função de Proteínas e Peptídeos, Universidade Estadual do Norte Fluminense Darcy Ribeiro, Campos dos Goytacazes, RJ, Brazil.
[3] Laboratório de Melhoramento e Genética Vegetal, Centro de Ciências e Tecnologias Agropecuárias, Universidade Estadual do Norte Fluminense Darcy Ribeiro, Campos dos Goytacazes, RJ, Brazil.

***Correspondence: Tel.: +55-22-27486490
E-mail address: valmg@uenf.com

```
                           1         10        20        30        40        50        60
CaDef1                     -----SKYFTGLCWTDSSCRKVCIEKDKFQDGHCSKIQRNCLCTK---------------------
IIFF7Ca (Q)                EICCQVPTTPFLCTNDPQ------------------------------------------------
IIFF7Ca (L)                EICCLVPTTPFLCTNDPQ------------------------------------------------
AHI85723.1                 EICCKVPTTPFLCTNDPQCKALCS-KVNYEDGHCFDILSKCVCMNRCVQDAKTLAAELLEEEFLKQ
AHI85724.1                 EICCKVPTTPFLCTNDPQCKALCS-KVNYEDGHCFDILSKCVCMNRCVQDAKTLAAELLEEEFLKQ
XP_016579689.1             EICCKVPTTPFLCTNDPQCKTLCS-KVNYEDGHCFDILSKCVCMNRCVQDAKTLAAELIEEEFLKQ
ABY66953.1                 EICCKVPTTPFLCTNDPQCKTLCS-KVNYEDGHCFDILSKCVCMNRCVQDAKTLAAELIEEEFLKQ
```

Supplementary figure. Alignment of amino acid residues from *Ca*Def1 and IIFF7Ca with other defensins and defensin-like peptides.

Alignment of the amino acid residues of *Ca*Def1 (red), previously described in Maracahipes et al. (2019), and the peptide IIFF7Ca (Q) and (L) obtained from F7 fraction correspondent to the 5 kDa band. Q and L forms of peptide correspond to the two different amino acids identified in

the same position, glutamine (Q) and leucine (L), respectively. These amino acid residues were underlined in the sequences. . Alignment was performed by Clustal Omega (https://www.ebi.ac.uk/Tools/msa/clustalo/; with default parameters). The sequences obtained were: Sequence ID: AHI85723.1 Stress-induced protein 18; Sequence ID: AHI85724.1 Stress-induced protein 19; Sequence ID: XP_016579689.1 flower defensin-like from *C. annuum*; and Sequence ID: ABY66953.1 Thionin-like. Note the alignment of the peptides *Ca*Def1 and IIFF7Ca in regard to the amino acids sequence. The differences observed indicate that the peptide obtained in this work is different from the defensin previously identified from the same fraction described in Maracahipes et al. (2019).
